# Combined treatment of transluminal Nd:YAG laser embolysis and hyperbaric oxygen for branch retinal artery occlusion

**DOI:** 10.3205/oc000163

**Published:** 2020-08-11

**Authors:** Hasim Uslu, Ayse Yagmur Kanra

**Affiliations:** 1 Hisar Hospital, Department of Ophthalmology, Istanbul, Turkey; 2 Sultanbeyli Dünyagöz Eye Hospital, Department of Ophthalmology, Istanbul, Turkey

**Keywords:** platelet-fibrin embolism, branch retinal artery occlusion, Nd:YAG laser embolism, hyperbaric oxygen treatment

## Abstract

**Objective:** To describe the clinical efficiency of transluminal Nd:YAG laser embolysis (TYE) and hyperbaric oxygen (HBO) as an off-label combined treatment for branch retinal artery occlusion (BRAO) with visible emboli.

**Methods:** A 77-year-old woman had a history of seeing a “shadow” in the lower visual field of the left eye for three days. Platelet-fibrin embolus at the arterial bifurcation was disintegrated by TYE technique and the patient was referred to HBO treatment for 20 sessions.

**Results:** One week after treatment, best-corrected visual acuity improved to 0.8 while a good arterial blood flow in the affected branch was seen. Platelet fibrin plaques had disappeared at fundus, and the pale appearance in the retina had decreased.

**Conclusions:** TYE and HBO combination treatment may be an effective and feasible treatment for restoration of blood flow and vision in BRAO cases caused by visible platelet-fibrin emboli.

## Introduction

Branch retinal artery occlusion (BRAO) may result from obstruction with platelet-fibrin emboli, cholesterol plaques or calcific emboli, typically located at the arterial bifurcations or areas of narrowing. Patients with BRAO present with sudden loss of vision. Fundus examination usually reveals a sectorial superficial whitening and a visible intralumenal embolus [1]. BRAO constitutes 38% of all acute retinal artery occlusions [2]. Even though there is no definitive treatment for BRAO, a number of treatment options are available, including carbogen inhalation, ocular massage, oral acetazolamide and topical anti-glaucomatous agents [3]. Opremcak and Benner indicated that transluminal Neodyium:yttrium-aluminum-garnet (Nd:YAG) laser embolysis (TYE) treatment resulted in immediate restoration of retinal blood flow and a good functional recovery [4]. On the other hand, hyperbaric oxygen (HBO) treatments for retinal artery obstructions have been performed and yielded favorable outcomes [5].

Due to the visual impairing nature of this condition, an effective emergency treatment capable of reversing the visual loss is needed. Considering prior studies, TYE and HBO as an off-label combined treatment was planned for a delayed BRAO case. Herein, we aim to report the clinical efficiency of TYE and HBO combined treatment for BRAO with visible emboli.

## Case description

**Figure 1:**
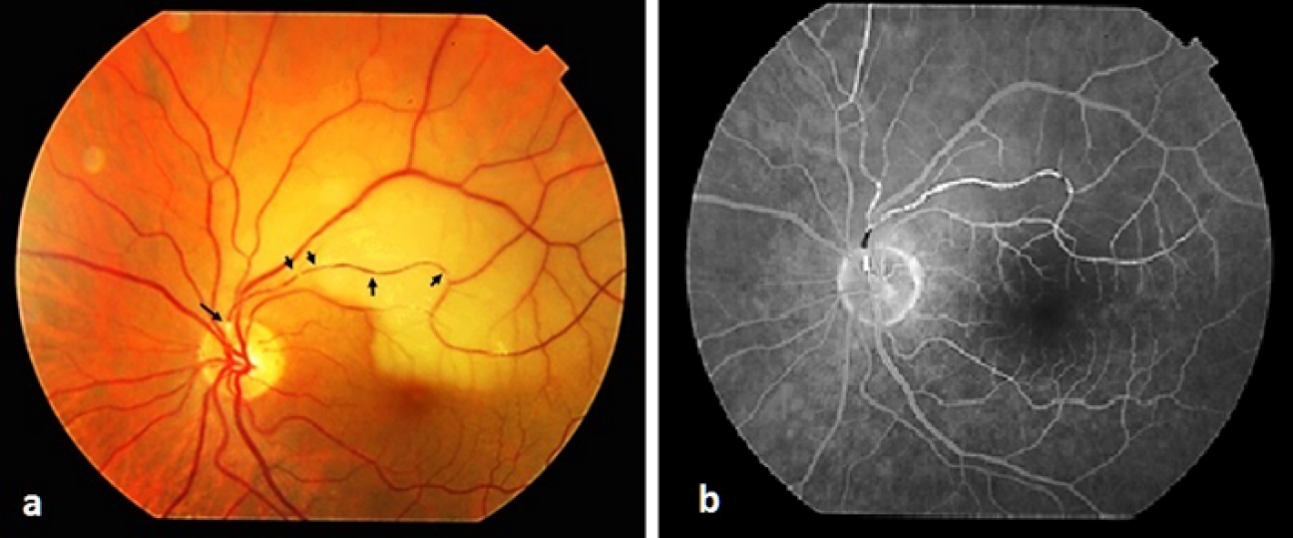
a) Color fundus photograph showing a fibrin-platelet embolus (arrow) and opacification of the retina along the superior-temporal vascular arcade; b) Fluorescein angiogram of the same patient demonstrating a blood flow defect in the affected superior temporal artery

**Figure 2:**
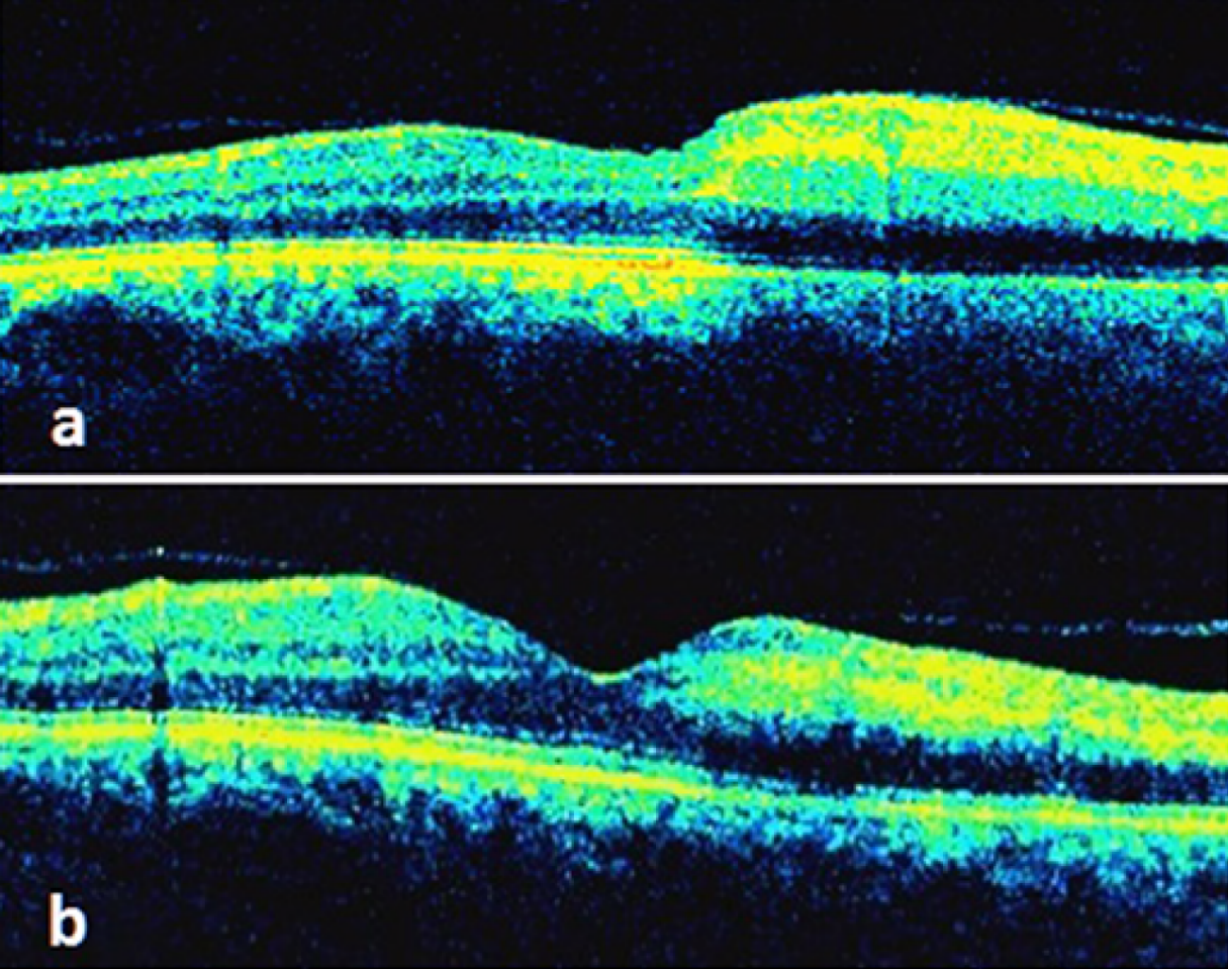
a) Optical coherence tomogram (OCT) demonstrating retinal edema in the superior temporal retinal artery region; b) OCT image shows improvements of retinal thickening one month following the combined treatment

**Figure 3:**
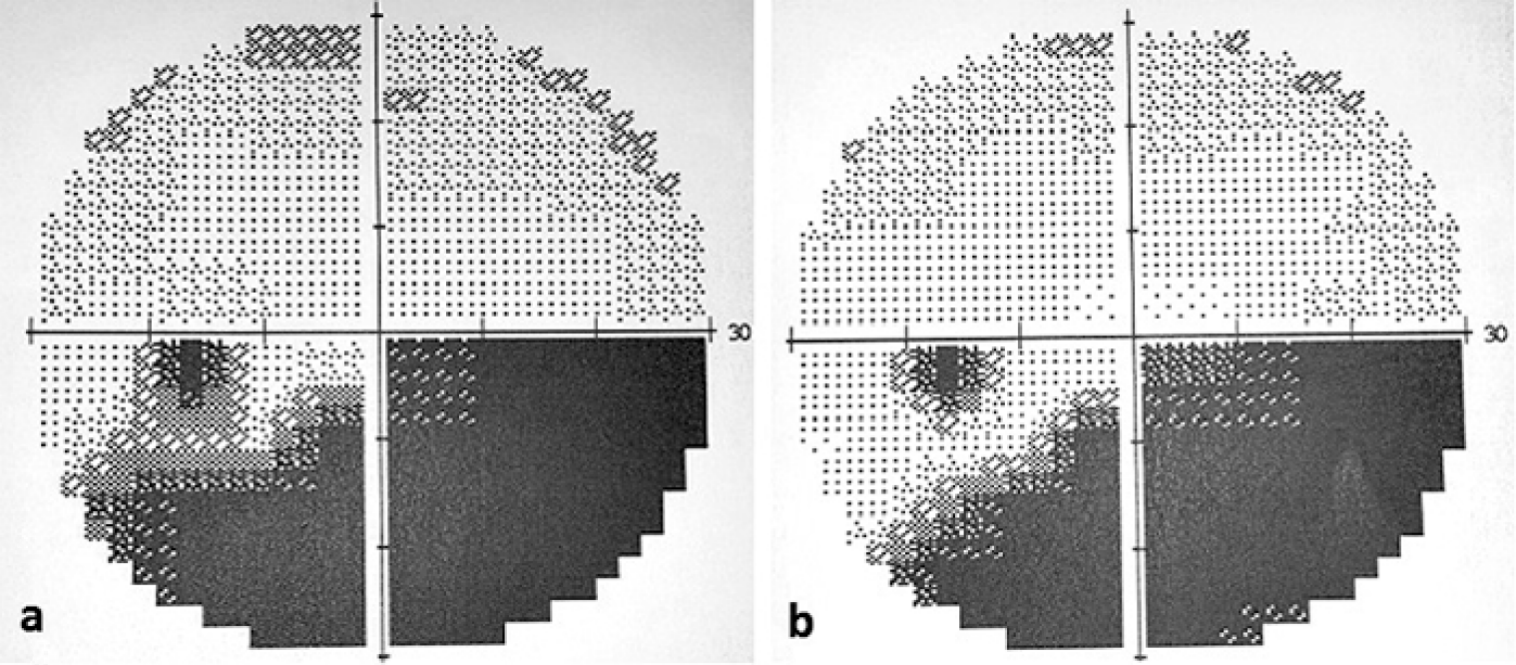
a) Visual field demonstrating inferior arcuate scotoma; b) Visual field after the combined treatment showing partial recovery of inferior arcuate scotoma

A 77-year-old woman presented to our clinic complaining of vision loss for three days in the inferior field of her left eye. She had well-controlled atrial fibrillation and hypertension in her systemic history. She also had a history of cataract surgery in both eyes with no complications. In the examination, best-corrected visual acuity (BCVA) was 0.9 in the right eye and 0.3 in the left eye on the Snellen scale. Intraocular pressure (IOP) was 14 mm Hg and 12 mm Hg in the right and left eye respectively, and both eyes were pseudophakic. Dilated fundus examination revealed a superior temporal artery branch occlusion with retinal paleness in the upper region of the macula, with a visible transluminal platelet-fibrin embolus at the arterial bifurcation, and numerous other small fibrin emboli scattered along the arterial branch (Figure 1a (Fig. 1)). Based on the signs obtained, the diagnosis was confirmed by fundus fluorescein angiography (FA), visual field (VF) testing (Humphrey 750i Visual Field Analyzer; Carl Zeiss Meditec, Inc., Dublin, CA, USA) and high definition-optic coherence tomography (HD-OCT) (Cirrus HD-OCT; Carl Zeiss Meditec, Dublin, CA, USA). FA showed delayed filling of the superotemporal artery and hypofluorescence in the surrounding artery (Figure 1b (Fig. 1)). HD-OCT revealed diffuse thickening of the inner retinal layers and increased reflectivity (Figure 2a (Fig. 2)), accompanied by a superior arcuate visual field defect in the VF testing (Figure 3a (Fig. 3)). Following initial ocular massage and application of anti-glaucomatous eye drops, cardiology consultation and carotid Doppler ultrasound were requested.

After obtaining detailed informed consent from the patient, TYE was applied directly to the arterial segment embedding the embolus [4]. The laser beam (Nd:YAG laser, YC-1800, Nidek Co., Ltd.) was focused on the embolus, using a Goldmann fundus contact lens. The laser energy level was started at 2.0 mJ pulse and increased by 1 mJ until embolysis (approximately 5 shots) was made at 4 mJ. During embolysis, a small amount of vitreous bleeding developed and was controlled by applying direct pressure over the eye with a contact lens. After the TYE procedure, hyperbaric oxygen treatment was started on the same day and continued for two sessions per day for two weeks, and then reduced to one session per day for a total of 20 sessions. Hyperbaric oxygen was given at 2.4 ATA (atmosphere absolute) pressures for 120 min in each session.

**Figure 4:**
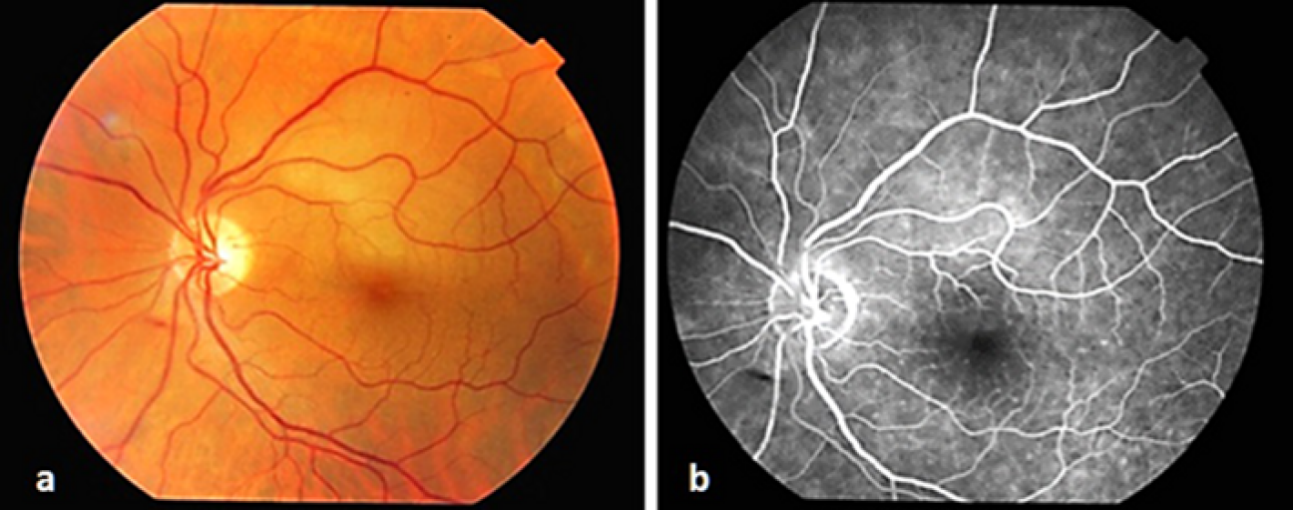
a) Color fundus photograph one week following TYE and HBO combined treatment; b) Fluorescein angiogram showing recovery of retinal blood flow

In the follow-up after one week, visual acuity of the patient improved to 0.8. In the fundus examination, no embolic material and hemorrhage was seen, and paleness in the upper temporal field of the macula had decreased (Figure 4a (Fig. 4)). In the follow-up FA, the normal blood flow was seen in the affected artery (Figure 4b (Fig. 4)), and the edema was noted to be regressed in the OCT image (Figure 2b (Fig. 2)). In the fourth week, BCVA was found 0.9. Humphrey 30-2 VF testing showed inferior arcuate scotoma (Figure 3a (Fig. 3)). The patients’s complaint of vision loss has disappeared completely. In the last examination done after 12 months, the patient’s visual acuity was 0.9, with inferior nasal quadrant scotoma (Figure 3b (Fig. 3)), no macular edema on OCT, normal appearance of the fundus and no visible edema.

## Discussion

Although a number of treatment options are available for BRAO, one kind of ocular emergency, there is no definitive treatment. Use of other systemic antifibrinolitic agents, intra-arterial fibrinolysis, surgical embolectomy or Nd:Yag laser embolysis (TYE) can be listed as other treatment options [6]. Some studies have reported hyperbaric oxygen as an effective treatment in retinal artery occlusions [5]. TYE has been used in the treatment of both central and branch retinal artery occlusion, and resulted in prompt visual improvement besides capillary reperfusion. Photodisruption of emboli has been found to be related with fast restoration of retinal blood flow and improvement in vision in these patients [4]. In our case, we detected that visual acuity increased by 6 lines (Snellen 0.8) in the first week with combined TYE and HBO treatment, and the blood flow has recovered completely in obstructed arteries in FA. BRAO generally leads to loss of vision with preserved central vision. In our patient, although central retinal involvement was not evident at the beginning, visual acuity was found to be subjectively low due to edema of the retina, older age, and shadowy vision. Retina affected by obstructed artery looks pale because of cloudy swelling that occurs due to intercellular edema, and ophthalmoscopic findings disappear with recovery of blood flow in the artery. Permanent intraretinal layer damage may lead to loss of visual field. It has been stated that a correlation was present between OCT images and histopathologic findings [7]. In our case, edema and pallor, although reduced, were still apparent in the retina field affected in the first month after TYE and HBO. Macular edema was compatible with OCT findings and visual field defect with preserved central vision. Hyperoxia caused by HBO treatment may induce recovery of the retina and macula oxygenation, and restrict fluid leak and edema via vasoconstriction [8]. It has been thought that retinal oxygen treatment can help keep retina cells alive by increasing the retinal oxygen level. In cats, it has been found that 70% oxygen given one day after experimental retinal detachment was highly effective to protect photoreceptors and reduce proliferation [9]. Johannes Menzel-Severing et al. have reported that 3 lines gain in visual acuity were attained after 3-month follow-up in patients who received hyperbaric oxygen treatment and hemodilution treatment together in central retinal artery occlusion, but a gain of one line visual acuity was achieved in the group without oxygen treatment [10]. In our patient, the initial BCVA was 0.3, and it was detected to be 0.8 at one-month follow-up.

## Conclusion

The results of our case revealed the satisfactory visual improvement following TYE and HBO treatment in patients with BRAO that have poor initial VA. Even if TYE and HBO combination treatment is an off-label modality, it may be considered in BRAO cases caused by visible platelet-fibrin emboli. It may be a fast, effective and feasible treatment for restoration of blood flow and vision.

## Notes

### Competing interests

The authors declare that they have no competing interests.

### Informed consent

Detailed informed consent has been obtained from the patient.
